# Intensive Care Unit Rotations and Predictors of Career Choice in Pulmonary/Critical Care Medicine: A Survey of Internal Medicine Residency Directors

**DOI:** 10.1155/2018/9496241

**Published:** 2018-03-06

**Authors:** Daniel J. Minter, Sean D. Levy, Sowmya R. Rao, Paul F. Currier

**Affiliations:** ^1^School of Medicine, University of California San Francisco, San Francisco, CA, USA; ^2^Department of Medicine, Division of Pulmonary, Critical Care, and Sleep Medicine, Beth Israel Deaconess Medical Center, Boston, MA, USA; ^3^Department of Surgery, Massachusetts General Hospital Biostatistics Center, Boston University, Boston, MA, USA; ^4^Department of Medicine, Division of Pulmonary, Critical Care, and Sleep Medicine, Massachusetts General Hospital, Boston, MA, USA

## Abstract

**Background:**

The United States (US) is experiencing a growing shortage of critical care medicine (CCM) trained physicians. Little is known about the exposures to CCM experienced by internal medicine (IM) residents or factors that may influence their decision to pursue a career in pulmonary/critical care medicine (PCCM).

**Methods:**

We conducted a survey of US IM residency program directors (PDs) and then used multivariable logistic regression to identify factors that were predictive of residency programs with a higher percentage of graduates pursuing careers in PCCM.

**Results:**

Of the 249 PDs contacted, 107 (43%) completed our survey. University-sponsored programs more commonly had large ICUs (62.3% versus 42.2%, *p*=0.05), primary medical ICUs (63.9% versus 41.3%, *p*=0.03), and closed staffing models (88.5% versus 41.3%, *p* < 0.001). Residents from university-sponsored programs were more likely to pursue specialty fellowship training (*p* < 0.001) overall but equally likely to pursue careers in PCCM as those from community-sponsored programs. Factors predictive of residencies with a higher percentage of graduates pursuing training in PCCM included larger ICUs (>20 beds), residents serving as code leaders, and greater proportion of graduates pursuing specialization.

**Conclusions:**

While numerous differences exist between the ICU rotations at community- and university-sponsored IM residencies, the percentage of graduates specializing in PCCM was similar. Exposure to larger ICUs, serving as code leaders, and higher rates of specialization were predictive of a career choice in PCCM.

## 1. Introduction

Since their advent during the polio epidemic of the mid-twentieth century, intensive care units (ICUs) and the specialty of critical care medicine (CCM) have become fundamental pillars in the care of critically ill patients [1]. Specialists in CCM, also referred to as “intensivists,” receive additional procedural and medical training and are ideally positioned to care for this increasingly complex population. The United States (US) is currently experiencing a worrisome shortage of intensivists, and the inability to meet staffing demands will likely continue to worsen as our population ages [2, 3]. Innovative ways to increase the supply of CCM trained physicians are now being explored.

Historically, intensivists in the US have largely been internal medicine (IM) residency graduates with subsequent training in pulmonary/critical care medicine (PCCM), along with surgeons and anesthesiologists [4]. More recently, trainees from other residencies (specifically emergency medicine and neurology), as well as IM graduates pursuing CCM fellowships without pulmonary training (IM-CCM), have begun entering the field in increasing numbers, with expanded board certification opportunities partially directed at increasing the supply of intensivists [3, 5, 6]. The largest pool of potential intensivist trainees, however, continues to be PCCM [7].

The Accreditation Council for Graduate Medical Education (ACGME) requires all graduates of IM residency programs to experience three to six months of dedicated critical care rotations during the 36 months of training but does not provide further guidance on how to structure such rotations [8]. A survey of university-affiliated PCCM fellowship directors about resident CCM education found that there was no standardized approach used by different programs [9]. Other studies of CCM education have largely focused on specific changes related to duty-hours or educational interventions [10–12].

Even less is known about how differences in CCM rotations influence the choice of IM graduates to pursue further training in PCCM. A survey study from 2002 found that IM residents harbored serious reservations about PCCM as a field, with heavy workloads and perceived stress among faculty and fellows being the most dissuading factors [13]. Conversely, residents cited the intellectual challenge, opportunity to manage critically ill patients, application of physiology, and ability to perform procedures as characteristics that increased their interest in the field. Other studies have investigated factors influencing career choice in a variety of specialties, with mentorship, intellectual challenge, and procedural opportunities being common themes [14–17].

In order to better inform future efforts to reform CCM education and increase interest in this specialty, we conducted a nationwide survey of US IM residency programs. Our primary objectives were to characterize the critical care rotations experienced by IM trainees at both community- and university-sponsored residency programs, as well as to identify aspects of the rotation that are predictive of IM graduates pursuing careers in PCCM.

## 2. Methods

### 2.1. Study Design and Population

A cross-sectional survey of IM residency PDs in the United States was conducted from February to March 2017. Participants included all directors of IM residency programs listed in the Electronic Residency Application Service (ERAS) for whom contact information was available online. An initial invitation letter was sent by email to all participants that provided an overview of the study objectives and methods. One week later, a link to the electronic survey was distributed, with biweekly reminders for up to 6 weeks. The protocol for this study was approved by the institutional review board at the Massachusetts General Hospital.

### 2.2. Surveys and Data Collection

Our survey instrument was modeled after the one developed by Almoosa et al. [9] and was subsequently modified with the author's permission. This revised survey primarily focused on the characteristics of medical ICU (MICU) rotations, CCM educational practices, ICU team structure, and fellowship choices. Prior to distribution, it was tested by multiple faculty members at the Massachusetts General Hospital for readability and appropriateness for the desired study population. The final survey (provided in the Supplementary Materials available here) was administered using Research Electronic Data Capture (REDCap) software.

### 2.3. Data Analysis

The survey data were categorized according to sponsorship (university versus community) and described using summary statistics (proportions, medians and interquartile ranges (IQR), or means and standard deviations). Categorical variables were compared using Fisher's exact test, while continuous variables were compared using two-sided *t*-tests and the nonparametric Kruskal–Wallis test. In order to evaluate for predictors of career choice in PCCM, we first classified programs as having a “high” or “low” percentage of the class pursuing training in PCCM (cutoff defined as the median percentage of a class going into PCCM). Adjusted odds ratios and 95% confidence intervals were obtained from a parsimonious multivariable logistic regression model to predict programs with a “high” percentage of the class pursuing training in PCCM and included variables considered to potentially influence the career choice of IM residents. Two-sided *p* values of ≤0.05 were considered to be significant. All analyses were conducted in STATA version 14 (Statacorp 2015, College Station, TX).

## 3. Results

### 3.1. Program Characteristics

Of the 249 PDs contacted, a total of 107 completed the survey (43%). Characteristics of the responding residency programs are displayed in Table 1. Sixty-one (57%) of the 107 programs were self-described as university-sponsored, and 46 (43%) were described as community-sponsored. The percentage of university-sponsored programs did not differ significantly between respondents and nonrespondents (67.29% versus 58.45%, *p*=0.19). University-sponsored residencies were more likely to be located in urban areas (80.3% versus 45.7%, *p* < 0.001) and have larger graduating classes (25.5 (18–38) versus 12 (9–16), *p* < 0.001). Residents in university-sponsored programs are exposed to a higher percentage of ICUs with greater than 20 beds (62.3% versus 42.2%, *p*=0.05), pure medical ICUs (63.9% versus 41.3%, *p*=0.03), and a closed ICU staffing model (88.5% versus 41.3%, *p* < 0.001).

While community-sponsored programs reported spending a slightly higher percentage of time teaching during rounds (33 (25–50) versus 27.5 (20–33), *p*=0.02), critical care education methods did not otherwise differ significantly. In both university- and community-sponsored programs, residents frequently served as code leaders (70.0% versus 71.7%, *p*=1.00) and performed a similar number of central lines by graduation (10 (IQR 6–12) versus 8 (IQR 5–17), *p*=0.25).

**Figure 1:**
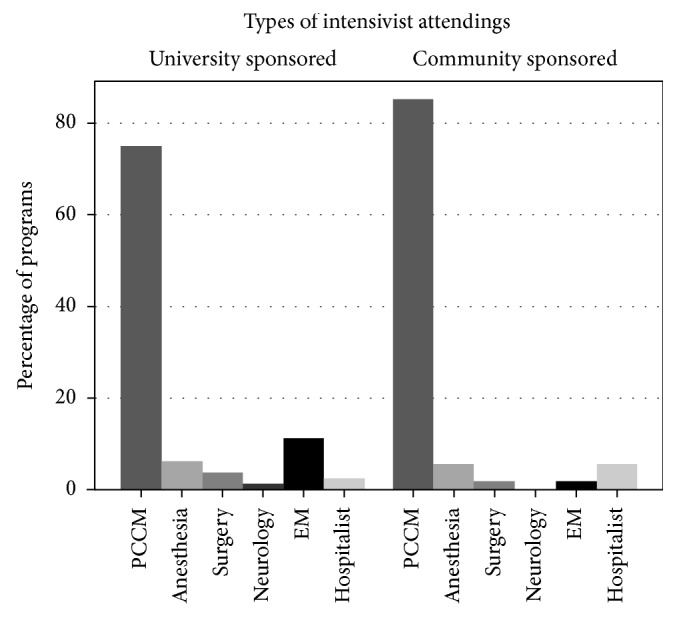
Bar graphs showing the percentage of residency programs reporting the presence of intensivists from different specialties on the ICU teaching service by residency sponsorship. In both groups, the most common intensivist specialty was PCCM, with the other base specialties variably represented in university- and community-sponsored programs. There was not a significant difference in the presence of non-PCCM attendings between the two types of residencies.

Few respondents reported having more than one attending per ICU team (16.7% versus 17.4%, *p*=1.00), while the presence of midlevel providers (50.0% versus 24.4%, *p*=0.01) and residents from other specialties (83.3% versus 35.6%, *p* < 0.001) as part of the ICU rounding team were more common in university-sponsored programs. The base specialties of intensivist attendings are presented in Figure 1.

### 3.2. Relationship of Variables to Percentage of Class Matching in PCCM

**Figure 2:**
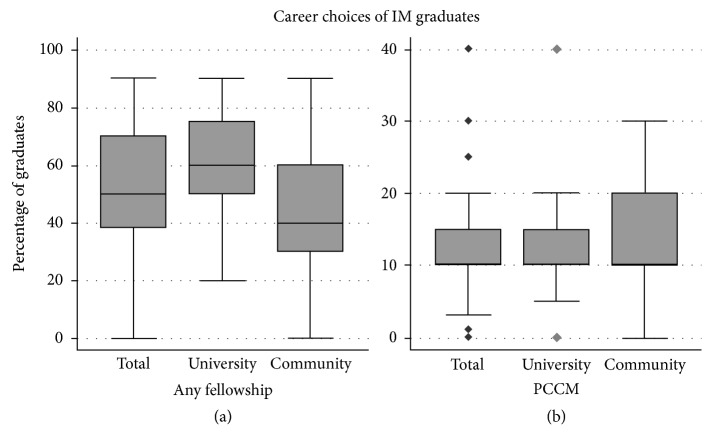
Box and whisker plots depicting the percentage of graduating IM residency classes pursuing any fellowship training (a) and PCCM (b) by residency sponsorship. A significantly higher median percentage of graduates from university-sponsored programs pursued any fellowship (60 (50–75) versus 40 (30–60), *p* < 0.001), but the class percentages pursuing PCCM specifically did not differ between residency types (10 (10–15) versus 10 (10–20), *p* < 0.64).

The career choices of graduating residents are shown in Figure 2. A higher median percentage of residents graduating from university-sponsored residencies go on to pursue any fellowship training than do those from community-sponsored programs (60 (50–75) versus 40 (30–60), *p* < 0.001), while the median percentage of the class pursuing fellowship training in PCCM was similar between the two groups (10 (10–15) versus 10 (10–20), *p*=0.64).

Table 2 shows the comparison of variables by whether the residency program had a “high” (above the median) or “low” (at or below the median) percentage of the graduating class pursuing fellowship training in PCCM. Programs with a high percentage of residents pursuing PCCM more frequently had ICUs with >20 beds (66.7% versus 44.1%, *p*=0.03) and lower percentages of residents pursuing any fellowship (17.2% versus 20.6%, *p* < 0.01).

### 3.3. Predictors of Programs with Higher PCCM Interest

Table 3 shows predictors for a higher/lower percentage of a class pursuing fellowship in PCCM in the final multivariable logistic regression model. Residents serving as hospital code leaders (OR 3.14 (1.01–9.82), *p*=0.05), the principle ICU having greater than 20 beds (3.32 (1.21–9.15), *p*=0.02), and the percentage of a class going into any fellowship (OR 1.07 (1.03–1.11), *p* < 0.001) predicted a higher percentage of the class pursuing training in PCCM after accounting for other variables.

## 4. Discussion

In this study, we surveyed US IM residency PDs, described the characteristics of the ICU rotation at both university- and community-sponsored residencies, and identified predictors of residency programs with higher percentages of graduates pursuing careers in PCCM. University- and community-sponsored residencies utilized similar educational methods and did not differ in the percentage of graduates specializing in PCCM. Programs with more graduates specializing in PCCM tended to have larger ICUs and lower rates of specialization. Residents serving as in-hospital code leaders, larger ICUs, and percentage of the class pursuing specialty training were identified as predictors of higher PCCM participation in our final multivariable logistic regression model.

Little has been published on the differences between community- and university-sponsored residency programs, and to our knowledge, this represents the first study to describe CCM education in both settings. While community programs tended to be less urban, have smaller classes, smaller ICUs, more mixed ICUs, and higher utilization of open or semiopen staffing patterns, there was not a significant difference in the percentage of graduates pursuing careers in PCCM. This suggests that these factors play little role, if any, in influencing career choice. In fact, our data suggest that, among those who do specialize, PCCM is a comparatively more common fellowship choice among residents at community-sponsored programs than their university-based peers. This may reflect a higher exposure to PCCM physicians than other subspecialists in community settings, as nearly all hospitals with training programs contain an ICU even if they lack other specialized services.

Our study also provided interesting insight into the changing staffing patterns of CCM. Both community- and university-sponsored programs reported having non-PCCM intensivists on the ICU teaching service, reflecting the increasing multidisciplinary nature of CCM as a specialty [3]. Notably, the presence of non-PCCM attending did not negatively influence residents' decision to specialize in PCCM, in contrast to prior studies that have emphasized the role of PCCM mentors in positively influencing resident perceptions of the specialty [13].

Finally, we have identified that exposure to larger ICUs, the opportunity to serve as in-hospital code leaders, and percentage of a class pursuing fellowship are predictors of resident career choice in PCCM. Exposure to larger ICUs may reflect a greater diversity of patient presentations, thereby increasing the intellectual challenge of the CCM rotation, a factor cited as one of the most attractive features of PCCM by IM graduates [13]. It might also reflect getting to experience more complex and innovative technologies (e.g., extracorporeal membrane oxygenation (ECMO)) and offer an opportunity to work with more skilled nurses and providers. Serving as the in-hospital code leader is often viewed as a stressful experience, for which residents may feel underprepared [18, 19]. Consequently, mastery of this skill may be perceived as a significant accomplishment by trainees and increase their interest in the care of critically ill patients. Some authors have noted a decline in the opportunity for residents to participate in in-hospital “code blues” in the era of quality improvement and resident work hour restrictions, highlighting the importance of designing residency education to maximize these valuable learning experiences [20]. Lastly, our regression model identified the percentage of a class that pursues specialization as predictive of higher PCCM interest when accounting for other covariates, but the magnitude of this association was weak (OR 1.07 (1.03–1.11), *p* < 0.001) (Table 2).

Our study had a few potential limitations. The response rate of 43%, while comparable to similar uncompensated studies of this medical education faculty [9, 12], was relatively low and thereby raises the possibility of response bias. While information was unavailable regarding the characteristics of the programs that failed to complete the survey, we found there to be a similar distribution of university- and community-sponsored programs among nonrespondents. Another important limitation of this study was the fact that the percentage of a class pursuing training in PCCM was estimated by PDs and therefore may be inaccurate. Previous studies on career choice, however, report similar percentages of IM residents planning to pursue PCCM fellowship training [21, 22].

Additionally, some have questioned whether expanding the number of PCCM fellowship positions is the best approach to address the shortage of intensivists, as PCCM physicians may spend a minority of their time practicing critical care [3, 4]. IM-CCM programs without a pulmonary component have drawn increased attention, since these practitioners spend a greater percentage of their clinical time in the ICU [4]. Unfortunately, our survey did not assess resident interest in this training pathway or exposure to IM-CCM mentors. However, this is unlikely to dramatically alter our conclusions, since IM-CCM training positions are dwarfed by those in PCCM (200 versus 1630, resp., in 2015-2016), and there has been a decline in the number of accredited IM-CCM fellowships in recent years [23].

Despite these limitations, this study adds valuable knowledge to the field of CCM education. Beyond better informing PDs regarding the diversity of ICU rotations nationwide, we identified easily implementable experiences (i.e., serving as hospital code leaders) that may increase resident interest in CCM. Modifications and improvements to residency critical care rotations could help to increase the intensivist supply, addressing an important public health concern.

## Figures and Tables

**Table 1:** Program and rotation characteristics.

	Total	University	Community	*p* value^ǂ^
*Residency/ICU characteristics*				
Urban location, *n* (%)	70 (65.4)	49 (80.3)	21 (45.7)	<0.001
Class size, median (IQR)	20 (12–30)	25.5 (18–38)	12 (9–16)	<0.001
ICU > 20 beds, *n* (%)	57 (53.8)	38 (62.3)	19 (42.2)	0.05
Primary MICU, *n* (%)	58 (54.2)	39 (63.9)	19 (41.3)	0.03
Closed ICU, *n* (%)	73 (68.2)	54 (88.5)	19 (41.3)	<0.001

*Education and the ICU rotation*				
Formal curriculum, *n* (%)	92 (86.0)	55 (90.2)	37 (80.4)	0.17
Online curriculum, *n* (%)	58 (57.4)	32 (57.1)	26 (57.8)	1.00
Extremely likely to receive formal training in invasive procedures, *n* (%)	62 (57.9)	37 (60.7)	25 (54.4)	0.56
Residents serve as code leaders, *n* (%)	75 (70.8)	42 (70.0)	33 (71.7)	1.000
Percent of time on rounds spent teaching, median (IQR)	30 (20–50)	27.5 (20–33)	33 (25–50)	0.02
Total months of ICU rotations, median (IQR)	5 (3–5)	5 (3.5–5.5)	4.25 (3–5)	0.71
Central lines performed, median (IQR)	10 (5–15)	10 (6–12)	8 (5–17.5)	0.25

*Team structure*				
More than one attending per team, *n* (%)	18 (17.0)	10 (16.7)	8 (17.4)	1.00
Programs with residents from other specialties, *n* (%)	66 (62.9)	50 (83.3)	16 (35.6)	<0.001
Programs with midlevels, *n* (%)	41 (39.1)	30 (50.0)	11 (24.4)	0.01
Programs with non-PCCM attendings, *n* (%)	23 (21.5)	15 (24.6)	8 (17.4)	0.48
Programs where PCCM most frequently serve as attendings, *n* (%)	100 (94.3)	56 (93.3)	44 (95.7)	0.70
Programs where PCCM are most heavily involved in education and mentorship, *n* (%)	100 (93.5)	55 (90.2)	45 (97.8)	0.24

^ǂ^ *p* values are based on two-sided Fisher's exact tests for categorical variables and Kruskal–Wallis tests for continuous variables.

**Table 2:** Different ICU characteristic in programs with low or high % of PCCM applicants.

	Low	High	*p* value^§^
University sponsored, *n* (%)	34 (57.6)	27 (58.7)	1.00
Urban, *n* (%)	36 (61.0)	33 (71.7)	0.30
Pure MICU, *n* (%)	33 (55.9)	25 (54.4)	1.00
Closed staffing, *n* (%)	39 (66.1)	34 (73.9)	0.40
ICU > 20 beds, *n* (%)	26 (44.1)	30 (66.7)	0.03
Formal curriculum, *n* (%)	51 (86.4)	39 (84.8)	1.00
Online curriculum, *n* (%)	27 (50)	30 (66.7)	0.11
Extremely likely to be trained in invasive procedures, *n* (%)	33 (55.9)	28 (60.9)	0.69
Serve as code leaders, *n* (%)	38 (64.4)	35 (77.8)	0.19
PCCM as specialty most involved in education/mentorship, *n* (%)	53 (89.8)	45 (97.8)	0.13
PCCM as the specialty that most frequently attends in the ICU, *n* (%)	53 (91.4)	45 (97.8)	0.22
Programs with non-PCCM attendings, *n* (%)	14 (23.7)	9 (19.6)	0.64
Midlevel providers on the ICU team, *n* (%)	21 (36.2)	20 (44.4)	0.42
Nonmedicine residents on the ICU team, *n* (%)	34 (57.6)	31 (70.5)	0.22
Total ICU months, mean (SD)	4.6 (2.4)	4.6 (1.2)	0.88
Class size, mean (SD)	21.8 (12.9)	23.1 (13.4)	0.63
Number of central lines, mean (SD)	10.1 (7.1)	13.0 (10.5)	0.10
Percent of class pursuing any fellowship, mean (SD)	47.9 (20.6)	59.3 (17.2)	<0.01

“High” and “low” refer to the percentage of the class entering PCCM with respect to the median percentage observed (10%); ^§^ *p* values are based on two-sided Fisher's exact tests for categorical variables and Students *t*-tests for continuous variables.

**Table 3:** Adjusted odds ratios (OR) and 95% confidence intervals (CI) from multivariable logistic regression model to predict residencies with higher % of graduates choosing PCCM as a career.

Covariates	OR	95% CI	*p* value^ǂ^
% of class pursuing fellowship	1.07	1.03–1.11	<0.001
ICU > 20 beds	3.33	1.21–9.15	0.02
Code leaders	3.14	1.01–9.82	0.05
Formal didactic curriculum	0.25	0.06–1.06	0.06
Primary MICU	0.37	0.12–1.16	0.09
University sponsorship	0.38	0.12–1.22	0.10
Central lines by graduation	1.04	0.99–1.10	0.13
Total months of ICU rotations	0.88	0.66–1.17	0.38
Non-PCCM faculty	1.59	0.51–4.97	0.42
Closed ICU	1.08	0.32–3.60	0.90

^ǂ^The model included all of the variables shown in the table.

## Abbreviations

ICU: Intensive care unit
MICU: Medical intensive care unit
CCM: Critical care medicine
IM: Internal medicine
PCCM: Pulmonary/critical care medicine
PD: Program director
ACGME: Accreditation Council for Graduate Medical Education
ERAS: Electronic Residency Application Service
IQR: Interquartile range
US: United States
ECMO: Extracorporeal membrane oxygenation.

## Supplementary Materials

Supplementary Materials The survey instrument used for data collection.
