# Supplementary material for: Intensive Care Unit Rotations and Predictors of Career Choice in Pulmonary/Critical Care Medicine: A Survey of Internal Medicine Residency Directors
Source: Crit Care Res Pract. 2018 Mar 6;2018:9496241. doi: 10.1155/2018/9496241 (PMC5859790; doi:10.1155/2018/9496241)
Supplement: Supplementary Materials — The survey instrument used for data collection. [file 9496241.f1.pdf]

Program Director Survey

Please complete the survey below.

Thank you!

GENERAL INFORMATION ABOUT THE RESIDENCY PROGRAM

Which of the following terms best describes your residency program? (choose one)
- ◯ University-sponsored or university-based
- ◯ Community-sponsored or community-based

Which of the following terms best describes your residency program? (choose one)
- ◯ Urban
- ◯ Suburban
- ◯ Rural

How many residents make up each graduating class? ______________________________

ICU DEMOGRAPHICS & RESOURCES

Please characterize your principle university-affiliated MEDICAL ICU
- ◯ MICU
- ◯ MICU/CCU
- ◯ MICU/SICU
- ◯ Other

If other, please describe. ______________________________

What best describes the staffing pattern of this ICU?
- ◯ Open
- ◯ Closed
- ◯ Semi-open (mandatory critical care consult on most/all patients)

How many beds are available in this ICU?
- ◯ Less than 10
- ◯ 10-20
- ◯ More than 20
- ◯ Don't know

How many months (on average) of dedicated ICU rotations do residents do during each year?

PGY-1 ______________________

PGY-2 ______________________

PGY-3 ______________________

EDUCATION METHODS & RESOURCES

Is there a faculty physician responsible for overseeing/organizing resident education in this ICU?
◯ Yes ◯ No ◯ Don't know

Do residents receive didactic training on standard ICU topics when they are rotating through this ICU?
◯ Yes ◯ No ◯ Don't know

Is there a core curriculum of ICU topics available online for the residents during this rotation?
◯ Yes ◯ No ◯ Don't know

Who does most of the resident teaching when they rotate through this ICU? (choose one)
◯ Attendings
◯ Fellows
◯ Residents themselves
◯ Other staff
◯ Everyone contributes equally
◯ It differs among rotations
◯ Don't know

Please check which of the following educational methods are used to teach residents when rotating through this ICU (Check all that apply).
☐ Didactic lectures
☐ Informal talks ("chalk-talks") in the ICU
☐ Bedside teaching during rounds
☐ Residents present topics
☐ Outside reading
☐ Simulated procedures training
☐ Audiovisual aids (e.g. slides)
☐ Other

How likely is it that residents receive formal training (either by simulation, bedside teaching, lectures) in invasive procedures (e.g. central line placement) during this ICU rotation?
◯ Extremely likely
◯ Very likely
◯ Very unlikely
◯ Extremely unlikely

On average, how many central lines do residents perform by graduation?
____

Do residents serve as hospital code leaders during their ICU rotations?
◯ Yes
◯ No
◯ Don't know

During the week, approximately how much time (in minutes) is spent every day teaching residents in this ICU?
____

Approximately what percent (%) of ICU rounds' time is spent on teaching? ________________________________

ICU TEAM STRUCTURE

At your institution, which intensivist attendings play a larger role in delivery of critical care medicine education and mentoring to the residents? (select one)
PCCM = Pulmonary/critical care medicine

- ◯ PCCM
- ◯ Non-PCCM faculty
- ◯ Roughly equal
- ◯ Don't know

How many attending physicians are there per ICU team?

- ◯ 1
- ◯ 2
- ◯ More than 2

What specialties of physicians serve as attendings on the ICU teaching services?

(choose all that apply)

- ☐ Pulmonary/CCM
- ☐ Anesthesia
- ☐ Surgery
- ☐ Neurology
- ☐ Emergency Medicine/CCM
- ☐ Hospital Medicine/Internal Medicine
- ☐ Other

What specialties of physicians spend the most time as attendings on the ICU teaching services?

- ◯ PCCM
- ◯ Non-PCCM faculty
- ◯ Roughly equal
- ◯ Don't know

Are midlevel providers (Nurse practitioners or physician's assistants) part of the ICU team?

- ◯ Yes
- ◯ No
- ◯ Don't know

Number of residents per team (on average)

PGY-1 ________________________________

PGY-2 ________________________________

PGY-3 ________________________________

Are residents/fellows from other specialties (i.e. anesthesia, surgical specialties) ever part of the rounding ICU team?

- ◯ Yes
- ◯ No
- ◯ Don't know

FELLOWSHIP CHOICES OF RESIDENTS

What percentage of graduating internal medicine residents go on to pursue subspecialty fellowship training? __________________________________

What percentage of graduating internal medicine residents go on to pursue a fellowship in pulmonary/critical care medicine? __________________________________
